# Supplementary material for: Three-year mortality in cryptococcal meningitis: Hyperglycemia predict unfavorable outcome
Source: PLoS One. 2021 May 28;16(5):e0251749. doi: 10.1371/journal.pone.0251749 (PMC8162582; doi:10.1371/journal.pone.0251749)
Supplement: S1 Table — (DOCX) [file pone.0251749.s001.docx]

**S1 Table. ICD diagnosis codes and medication for comorbidities used in this study.**

| Comorbidity | ICD-9-CM |  | Medication |
| --- | --- | --- | --- |
| Human immunodeficiency virus | 42-44 |  | Not applicable |
| Organ transplantation | V42 |  | Not applicable |
| Malignancy | 140-208 |  | Not applicable |
| Diabetes mellitus | 250 | and | Insulin, Oral hypoglycemic drugs |
| Autoimmune disease | 710, 714, 720, 725-729 |  | Not applicable |
| Liver cirrhosis | 571 |  | Not applicable |
| Chronic kidney disease | 585 |  | Not applicable |
