# Supplementary material for: Three-year mortality in cryptococcal meningitis: Hyperglycemia predict unfavorable outcome
Source: PLoS One. 2021 May 28;16(5):e0251749. doi: 10.1371/journal.pone.0251749 (PMC8162582; doi:10.1371/journal.pone.0251749)
Supplement: S2 Table — (DOCX) [file pone.0251749.s002.docx]

**S2 Table. Cryptococcal species that grew in the cerebrospinal fluid.**

| Pathogens | N | % |
| --- | --- | --- |
| *Cryptococcus neoformans* | 63 | 75.9 |
| *Cryptococcus neoformans_var_grubii* | 17 | 20.5 |
| *Cryptococcus neoformans_var_grubii*, *Cryptococcus neoformans* | 1 | 1.2 |
| *Cryptococcus gattii* | 1 | 1.2 |
| *Cryptococcus spp.* | 1 | 1.2 |
