# Supplementary material for: Three-year mortality in cryptococcal meningitis: Hyperglycemia predict unfavorable outcome
Source: PLoS One. 2021 May 28;16(5):e0251749. doi: 10.1371/journal.pone.0251749 (PMC8162582; doi:10.1371/journal.pone.0251749)
Supplement: S3 Table — (DOCX) [file pone.0251749.s003.docx]

**S3 Table. Demographic and clinical characteristics of patients with Cryptococcal meningitis during 2003 – 2016 at China Medical University Hospital.**

| **Variables^a^** | **Overall** | **HIV** | **Non HIV** | **P-value^b^** |
| --- | --- | --- | --- | --- |
| **N** | **N = 83** | **N = 33** | **N = 50** |  |
| Age at index date^c^ (year) | 49 (34.5, 64.5) | 34 (30, 39) | 59 (49.2, 72) | < 0.001 |
| Male | 64 (77.1) | 32 (97) | 32 (64) | 0.001 |
| **Comorbidities^d^** |  |  |  |  |
| Organ transplantation | 2 (2.4) | 0 (0) | 2 (4) | 0.515 |
| Malignancy | 8 (9.6) | 1 (3) | 7 (14) | 0.137 |
| Diabetes mellitus | 9 (10.8) | 0 (0) | 9 (18) | 0.01 |
| Autoimmune disease | 5 (6) | 0 (0) | 5 (10) | 0.152 |
| Liver cirrhosis | 10 (12) | 0 (0) | 10 (20) | 0.005 |
| Chronic kidney disease | 5 (6) | 0 (0) | 5 (10) | 0.152 |
| None of above | 53 (63.9) | 32 (97.0) | 21 (42.0) | < 0.001 |
| **Corticosteroid history within 90 days prior to the index date** |  |  |  |  |
| Cortisone acetate | 2 (2.4) | 0 (0) | 2 (4) | 0.515 |
| Dexamethasone | 5 (6) | 1 (3) | 4 (8) | 0.644 |
| Prednisolone | 8 (9.6) | 0 (0) | 8 (16) | 0.019 |
| **Antiretroviral therapy within 1 year prior to the index date** | 11 (13.3) | 11 (33.3) | 0 (0) | < 0.001 |
| **Symptoms, n (%)** |  |  |  |  |
| Fever | 41 (50) | 19 (57.6) | 22 (44.9) | 0.324 |
| Headache | 48 (58.5) | 23 (69.7) | 25 (51) | 0.121 |
| Neck stiffness | 7 (8.5) | 5 (15.2) | 2 (4.1) | 0.108 |
| Altered Mental Status | 33 (40.2) | 7 (21.2) | 26 (53.1) | 0.01 |
| Visual symptoms | 9 (11) | 2 (6.1) | 7 (14.3) | 0.306 |
| Auditory symptoms | 8 (9.8) | 1 (3) | 7 (14.3) | 0.137 |
| Seizures | 9 (11) | 4 (12.1) | 5 (10.2) | 1 |
| Glasgow Coma Scale within - /+3 days of the index date | 15 (10, 15) | 15 (15, 15) | 10 (8, 15) | 0.001 |
| **Lumbar puncture during hospitalization** |  |  |  |  |
| First open pressure (cmH_2_O) | 24 (16, 29.2) | 24.5 (18.9, 28) | 23.5 (16, 30.5) | 1 |
| **Profiles of CSF within -/+ 30 days of the index date** |  |  |  |  |
| Positive *Cryptococcal* antigen | 73 (88.0) | 42 (84.0) | 31 (93.9) | 0.302 |
| Positive India Ink | 66 (79.5) | 29 (87.9) | 37 (74.0) | 0.209 |
| White blood cell (/ul) | 47 (5, 156) | 10 (2, 83) | 85.5 (9.2, 261.2) | 0.003 |
| Red blood cell (/ul) | 23 (3, 155) | 16 (2, 82) | 26 (3, 162.5) | 0.514 |
| Total protein (mg/dL) | 92.8 (53.5, 189.1) | 70 (46, 125) | 136.1 (69, 228) | 0.006 |
| CSF glucose (mg/dL) | 39 (16, 53.5) | 48 (34, 58) | 27.5 (12, 47.5) | 0.009 |
| **Positive blood culture that grew *Cryptococci*** | 29 (34.9) | 18 (54.5) | 11 (22.0) | 0.005 |
| **Serum biochemical profiles within 30 days prior to the index date** |  |  |  |  |
| Positive *Cryptococcal* antigen | 61 (73.5) | 26 (78.8) | 35 (70.0) | 0.526 |
| Serum glucose (mg/dL) | 122.5 (109.8, 166.5) | 113 (98, 138) | 133 (112.8, 185.2) | 0.005 |
| ＜140 mg/dL | 50 (60.2) | 24 (72.7) | 26 (52.0) | < 0.001 |
| 140-199 mg/dL | 17 (20.5) | 6 (18.2) | 11 (22.0) |  |
| ≧200 mg/dL | 15 (18.1) | 2 (6.1) | 13 (26.0) |  |
| ESR (mm/hr) | 31 (16, 61) | 51 (19.5, 66.5) | 21 (14, 47) | 0.27 |
| White blood cell (1x10^3^/ul) | 8 (5.8, 10.9) | 6.5 (4.3, 9.1) | 9.1 (6.5, 13.8) | 0.005 |
| Creatinine (mg/dL) | 0.9 (0.7, 1) | 0.8 (0.6, 1) | 0.9 (0.7, 1.1) | 0.098 |
| Alanine aminotransferase (ALT) (IU/L) | 26.5 (18.2, 50.8) | 25 (18.8, 55) | 27 (17.5, 49) | 0.966 |
| Hemoglobin (g/dL) | 11.9 (10.1, 13.3) | 12.5 (11.7, 13.9) | 10.9 (9.7, 12.8) | 0.008 |
| CD4 / mm^3^ | 34.5 (19.5, 67.5) | 30 (17.5, 62) | 82 (55.5, 533.5) | 0.156 |
| **Medical treatment during hospitalization** |  |  |  |  |
| Amphotericin B or Liposomal Amphotericin B | 68 (81.9) | 29 (87.9) | 39 (78) | 0.394 |
| Amphotericin B | 64 (77.1) | 29 (87.9) | 35 (70) | 0.103 |
| Liposomal Amphotericin B | 20 (24.1) | 6 (18.2) | 14 (28) | 0.446 |
| Flucytosine | 60 (72.3) | 24 (72.7) | 36 (72) | 1 |
| Fluconazole | 76 (91.6) | 30 (90.9) | 46 (92) | 1 |
| Itraconazole | 1 (1.2) | 1 (3) | 0 (0) | 0.398 |
| **Surgical shunt** | 25 (30.1) | 10 (30.3) | 15 (30) | 1 |
| **Outcome** |  |  |  |  |
| **Mortality, n (%)** |  |  |  |  |
| 1-month | 14 (16.9) | 2 (6.1) | 12 (24.0) | 0.066 |
| 3-month | 22 (26.5) | 4 (12.1) | 18 (36.0) | 0.031 |
| 1-year | 38 (45.8) | 8 (24.2) | 30 (60.0) | 0.003 |
| 3-year | 45 (54.2) | 10 (30.3) | 35 (70.0) | < 0.001 |

**Abbreviations: CSF, cerebrospinal fluid; ESR, erythrocyte sedimentation rate; IQR, interquartile range.**

a Categorical variables are presented as frequency (%) and continuous variables are presented as median (IQR), if not otherwise specified.

b P-values are calculated by Kruskal-Wallis test for continuous variables and Chi-square test (or Fisher’s exact test as appropriate) for categorical variables.

c Index date was the date of positive cerebrospinal fluid culture that grew Cryptococcal species.

d Comorbidities were defined by the ICD diagnosis codes corresponding to each disease (Supplementary Table 1) that were recorded within one year prior to the index date.
