# Supplementary material for: Three-year mortality in cryptococcal meningitis: Hyperglycemia predict unfavorable outcome
Source: PLoS One. 2021 May 28;16(5):e0251749. doi: 10.1371/journal.pone.0251749 (PMC8162582; doi:10.1371/journal.pone.0251749)
Supplement: Attachment — Submitted filename: response to reviewers 0410.docx [file pone.0251749.s004.docx]

**Reply to Reviewers’ Comments**

PONE-D-20-37177

Three-year Mortality in Cryptococcal Meningitis: Hyperglycemia Predict Unfavorable Outcome

**Reviewer #1**

In this manuscript, Tsai et. al. evaluated prognostic factors for long-term survival for cryptococcal meningitis. The authors evaluated 3-year mortality in a retrospective cohort study with 83 confirmed CM patients in China Medical University Hospital. Their analysis showed that advanced age, human immunodeficiency virus (HIV) seronegative state, low Glasgow Coma Scale score on admission, decreased hemoglobin and hyperglycemia on diagnosis were associated with 3-year mortality. After multivariate adjustment, only severe hyperglycemia was associated with 3-year mortality. The reviewer has several concerns about the study:

1. **The introduction can be strengthened if the authors discuss what is previously known about predictive factors of short and long-term mortality and why a new long-term mortality analysis is needed.**

**Reply**

Thank you for your suggestions. We have revised our introduction as followed (Please see below or the revised manuscript)

In Introduction (page 3, paragraph 1, lines 7-19 in the revised manuscript)

~~Several studies emphasizing on the predictive factors of short-term mortality have shown varied results [5,6].~~ Previous studies suggested that older age, altered neurological status, and high fungal burden were risk factors for 3-month to 1-year mortality in patient with CM. In addition, lacking of antiretroviral therapy (ART) and low CD4 cell counts at induction therapy phase predicted poor prognosis specifically for HIV patients with CM. By contrast, delayed diagnosis and coexistence of underlying comorbidities were related to high mortality in non-HIV patients with CM.

Current therapeutic guideline recommended an at least one-year pharmacological treatment program for HIV patients with CM. However, a survey of prognostic factors for more than 1 year ~~long-term~~ outcomes was still lacking.

2. **it is unclear whether prognostic factors predict long-term mortality analysis also predict short-term mortality. The authors should discuss if such differences exist.**

**Reply-**

We appreciate the insightful comments and have done the 3-month mortality analysis (Table R1). In our cohort, the 3-month mortality was 27% (22 deaths among 83 patients). The prognostic factors for unfavorable outcomes in 3-month and 3-year were quite similar (advanced age, low Glasgow Coma Scale on admission, non-HIV state, decreased hemoglobin on diagnosis and hyperglycemia on diagnosis). We have also found that increased blood WBC level and decreased CD4 cell counts might be related to 3-month mortality (Table R1). However, these effects faded after fitting to univariate and multivariate Cox proportional hazards models with age as the time scale. The risk of 3-month mortality associated with hyperglycemia was observed but this association was not statistically significant (Table R2). We apologized that we did not provide 3-month mortality analysis in our manuscript because of small sample size.

**Table R1. Demographic and clinical characteristics of patients with Cryptococcal meningitis during 2003 – 2016 at China Medical University Hospital.**

| **Variables^a^** | **Overall** | **Died within 3 months** | **Alive within 3 months** | **P-value^b^** |
| --- | --- | --- | --- | --- |
| **N** | **N = 83** | **N = 22** | **N = 61** |  |
| Age at index date^c^ (year) | 49 (34.5, 64.5) | 60 (52.2, 70.5) | 40 (31, 57) | 0.001 |
| Male | 64 (77.1) | 15 (68.2) | 49 (80.3) | 0.386 |
| **Comorbidities^d^** |  |  |  |  |
| Human immunodeficiency virus | 33 (39.8) | 4 (18.2) | 29 (47.5) | 0.031 |
| Diabetes mellitus | 9 (10.8) | 4 (18.2) | 5 (8.2) | 0.236 |
| Hypertension | 15 (18.1) | 6 (27.3) | 9 (14.8) | 0.208 |
| Malignancy | 8 (9.6) | 1 (4.5) | 7 (11.5) | 0.675 |
| Organ transplantation | 2 (2.4) | 2 (9.1) | 0 (0.0) | 0.068 |
| Autoimmune disease | 5 (6) | 0 (0.0) | 5 (8.2) | 0.318 |
| Liver cirrhosis | 10 (12) | 7 (31.8) | 3 (4.9) | 0.003 |
| Non-infectious kidney disease | 5 (6) | 2 (9.1) | 3 (4.9) | 0.605 |
| **Human immunodeficiency virus medication history within 365 days prior to the index date** | 11 (13.3) | 2 (9.1) | 9 (14.8) | 0.719 |
| **Corticosteroid history within 90 days prior to the index date** |  |  |  |  |
| Cortisone acetate | 2 (2.4) | 1 (4.5) | 1 (1.6) | 0.462 |
| Dexamethasone | 5 (6) | 2 (9.1) | 3 (4.9) | 0.605 |
| Prednisolone | 8 (9.6) | 0 (0.0) | 8 (13.1) | 0.102 |
| **Symptoms, n (%)** |  |  |  |  |
| Fever | 41 (50) | 14 (66.7) | 27 (44.3) | 0.190 |
| Headache | 48 (58.5) | 10 (47.6) | 38 (62.3) | 0.263 |
| Neck stiffness | 7 (8.5) | 2 (9.5) | 5 (8.2) | 1.000 |
| Altered Mental Status | 33 (40.2) | 14 (66.7) | 19 (31.1) | 0.016 |
| Visual symptoms | 9 (11) | 1 (4.8) | 8 (13.1) | 0.433 |
| Auditory symptoms | 8 (9.8) | 1 (4.8) | 7 (11.5) | 0.675 |
| Seizures | 9 (11) | 3 (14.3) | 6 (9.8) | 0.693 |
| Glasgow Coma Scale within - /+3 days of the index date | 15 (10, 15) | 9 (6, 11) | 15 (13.8, 15) | 0.001 |
| **Lumbar puncture during hospitalization** |  |  |  |  |
| First open pressure (cmH2O) | 24 (16, 29.2) | 24 (17, 26) | 24 (16.1, 35.2) | 0.578 |
| **Profiles of CSF within -/+ 30 days of the index date** |  |  |  |  |
| Positive *Cryptococcal* antigen | 73 (88.0) | 17 (80.0) | 56 (90.0) | 0.120 |
| Positive India Ink | 66 (79.5) | 19 (90.0) | 47 (80.0) | 0.539 |
| CSF routine |  |  |  |  |
| White blood cell (/ul) | 47 (5, 156) | 65 (41, 220) | 29 (5, 152) | 0.140 |
| Red blood cell (/ul) | 23 (3, 155) | 52 (8.2, 230) | 16 (3, 100) | 0.243 |
| Total protein (mg/dL) | 92.8 (53.5, 189.1) | 104.5 (58.5, 183.7) | 86.5 (51.8, 190.1) | 0.806 |
| CSF glucose (mg/dL) | 39 (16, 53.5) | 22.5 (9, 48) | 40 (18, 58) | 0.105 |
| **Positive blood culture that grew *Cryptococci*** | 29 (34.9) | 8 (36.4) | 21 (34.4) | 1.000 |
| **Serum biochemical profiles within 30 days prior to the index date** |  |  |  |  |
| Positive *Cryptococcal* Antigen | 61 (73.5) | 17 (80.0) | 44 (70.0) | 0.852 |
| Serum glucose (mg/dL) | 122.5 (109.8, 166.5) | 179.5 (148.2, 216) | 113 (106.2, 133.5) | < 0.001 |
| ＜140 mg/dL | 50 (60.2) | 4 (18.2) | 46 (75.4) | <0.001 |
| 140-199 mg/dL | 17 (20.5) | 9 (40.9) | 8 (13.1) |  |
| ≧200 mg/dL | 15 (18.1) | 9 (40.9) | 6 (9.8) |  |
| ESR (mm/hr) | 31 (16, 61) | 27 (13.5, 62.2) | 31 (17, 60) | 0.665 |
| White blood cell (1x10^3^/ul) | 8 (5.8, 10.9) | 9.2 (7.3, 14.6) | 7.2 (5.4, 10.1) | 0.014 |
| Creatinine (mg/dL) | 0.9 (0.7, 1) | 1 (0.8, 1.3) | 0.8 (0.7, 1) | 0.059 |
| Alanine aminotransferase (ALT) (IU/L) | 26.5 (18.2, 50.8) | 27 (21.5, 82.8) | 24 (17.8, 46.2) | 0.300 |
| Hemoglobin (g/dL) | 11.9 (10.1, 13.3) | 10.5 (9.5, 10.9) | 12.5 (10.8, 13.7) | 0.002 |
| CD4 / mm^3^ | 34.5 (19.5, 67.5) | 16 (9, 17.5) | 42 (24.5, 74.5) | 0.038 |
| **Medical treatment during hospitalization** |  |  |  |  |
| Amphotericin B or Liposomal Amphotericin B | 68 (81.9) | 17 (77.3) | 51 (83.6) | 0.528 |
| Amphotericin B | 64 (77.1) | 17 (77.3) | 47 (77) | 1 |
| Liposomal Amphotericin B | 20 (24.1) | 5 (22.7) | 15 (24.6) | 1 |
| Flucytosine | 60 (72.3) | 16 (72.7) | 44 (72.1) | 1 |
| Fluconazole | 76 (91.6) | 18 (81.8) | 58 (95.1) | 0.076 |
| Surgical shunt | 25 (30.1) | 5 (22.7) | 20 (32.8) | 0.541 |

**Abbreviations: CSF, cerebrospinal fluid; ESR, erythrocyte sedimentation rate; IQR, interquartile range.**

a Categorical variables are presented as frequency (%) and continuous variables are presented as median (IQR), if not otherwise specified.

b P-values are calculated by Kruskal-Wallis test for continuous variables and Chi-square test (or Fisher’s exact test as appropriate) for categorical variables.

c Index date was the date of positive cerebrospinal fluid culture that grew Cryptococcal species.

d Comorbidities were defined by the ICD diagnosis codes corresponding to each disease (Supplementary Table 1) that were recorded within one year prior to the index date.

**Table R2. Univariate and multivariate Cox proportional hazards analysis for the outcome of 3-month mortality.**

|  | Univariate | | Multivariate | |
| --- | --- | --- | --- | --- |
| Variables | Crude HR  (95% CI) | P-value | Adjusted HR  (95% CI) | P-value |
| Glasgow Coma Scale | 1.048 (0.81,1.36) | 0.720 | 1.008 (0.64,1.6) | 0.973 |
| Human Immunodeficiency Virus | 473159735.756 (0,Inf) | 0.999 | 158244434.815 (0,Inf) | 0.999 |
| Hemoglobin (g/dL) | 0.961 (0.64,1.45) | 0.846 | 1.113 (0.57,2.17) | 0.750 |
| 140≦Glucose<200 (Ref. Glucose<140) | 5.659 (0.61,52.71) | 0.126 | 4.669 (0.23,93.52) | 0.309 |
| Glucose≧200 (Ref. Glucose<140) | 8.662 (0.66,114.57) | 0.100 | 8.36 (0.43,161.53) | 0.157 |
| White blood cell (1x103/ul) | 1.011 (0.91,1.12) | 0.823 |  |  |
| CD4 / mm^3^ | 0.992 (0.95,1.03) | 0.684 |  |  |

Abbreviations: HR, hazard ratio.

*: P < 0.05

3. **Can the authors analyze or discuss if similar prognostic factors predict mortality for CM patients that are immune-suppressed (due to HIV/treatments) or immunocompetent ?**

**Reply**

We thank the reviewer for the suggestion. We have done the subgroup analysis for HIV status in 3-year mortality (Table R3). Unfortunately, the sample size was too small for assessing the mortality risk. In non-HIV group, we could not find statistical significance in any variables, but hyperglycemia showed the trend for unfavorable outcome prediction.

**Table R3. Univariate and multivariate Cox proportional hazards analysis for the outcome of 3-Year mortality.**

|  | **Human Immunodeficiency Virus** | | **Non-** **Human Immunodeficiency Virus** | |  |
| --- | --- | --- | --- | --- | --- |
| Variables | Crude HR  (95% CI) | P-value | Adjusted HR  (95% CI) | P-value | P for interaction for Glucose and HIV |
|  |  |  |  |  |  |
| Glasgow Coma Scale | 16088.274 (0,Inf) | 0.999 | 0.996 (0.82,1.21) | 0.970 | Cannot be estimated. |
| Hemoglobin | 1.189 (0.52,2.72) | 0.671 | 0.898 (0.64,1.26) | 0.529 |  |
| 140≦Glucose<200 (Ref. Glucose<140) | 4.105 (0.25,67.13) | 0.310 | 1.269 (0.23,6.97) | 0.780 |  |
| Glucose≧200 (Ref. Glucose<140) | - | - | 4.774 (0.79,28.82) | 0.087 |  |

**Reviewer #2**

The authors of this manuscript performed a retrospective study of 83 patients with confirmed cryptococcal meningitis (CM). One overall conclusion is that severe hyperglycemia is a better predictor of 3-yr mortality in CM patients than comorbidity of diabetes mellitus. However, one important point that deserves more detailed discussion is the lower mortality rate in CM patients with HIV when compared to CM patients without HIV infection. The manuscript would benefit from a more detailed analysis of the differences between these two groups of patients. If the data is available, it would be very interesting to know the differences in age distribution, presence of comorbidities and some discussion if HIV treatment itself had any effect on CM mortality in CM patients with HIV infection. While the study is limited in its sample size, the authors have performed sufficient comparison and statistical analysis to reach their conclusions.

**Reply**

Thank you so much for your suggestions.

1. We tried to identify the percentage of HIV patient who have been accepted antiretroviral therapy within 1 year prior to the CM diagnosis. We also tried to find an otherwise “normal” group without any immunocompromised status. We have modified our table 1 and revised our discussion as following (please see the revised manuscripts, page 12, paragraph 2, line 5-12)

The use of effective antiretroviral therapy for HIV-infected patients contributed to the decline of cryptococcosis incidence. In our cohort, the percentage of previous use of antiretroviral therapy in HIV-infected CM patients was 33.3% (11 of 33 patients). For HIV-seronegative patients, the host immune status was heterogeneous. Our study identified 25.3% (21 of 83 patients) otherwise “normal” patients without comorbidities including organ transplantation, malignancy, diabetes mellitus, autoimmune disease, liver cirrhosis or chronic kidney disease. This phenotypically “normal” group probably represented the congruence of subclinical innate or acquired immunodeficiencies.

2. We provided a table for HIV-infected and non-HIV infected groups comparison. We apologized that the sample size was too small for further analysis. (Table R4; same as Supplementary table 3)

**Table R4. Demographic and clinical characteristics of patients with Cryptococcal meningitis during 2003 – 2016 at China Medical University Hospital.**

| **Variables^a^** | **Overall** | **HIV** | **Non HIV** | **P-value^b^** |
| --- | --- | --- | --- | --- |
| **N** | **N = 83** | **N = 33** | **N = 50** |  |
| Age at index date^c^ (year) | 49 (34.5, 64.5) | 34 (30, 39) | 59 (49.2, 72) | < 0.001 |
| Male | 64 (77.1) | 32 (97) | 32 (64) | 0.001 |
| **Comorbidities^d^** |  |  |  |  |
| Organ transplantation | 2 (2.4) | 0 (0) | 2 (4) | 0.515 |
| Malignancy | 8 (9.6) | 1 (3) | 7 (14) | 0.137 |
| Diabetes mellitus | 9 (10.8) | 0 (0) | 9 (18) | 0.01 |
| Autoimmune disease | 5 (6) | 0 (0) | 5 (10) | 0.152 |
| Liver cirrhosis | 10 (12) | 0 (0) | 10 (20) | 0.005 |
| Chronic kidney disease | 5 (6) | 0 (0) | 5 (10) | 0.152 |
| None of above | 53 (63.9) | 32 (97.0) | 21 (42.0) | < 0.001 |
| **Corticosteroid history within 90 days prior to the index date** |  |  |  |  |
| Cortisone acetate | 2 (2.4) | 0 (0) | 2 (4) | 0.515 |
| Dexamethasone | 5 (6) | 1 (3) | 4 (8) | 0.644 |
| Prednisolone | 8 (9.6) | 0 (0) | 8 (16) | 0.019 |
| **Antiretroviral therapy within 1 year prior to the index date** | 11 (13.3) | 11 (33.3) | 0 (0) | < 0.001 |
| **Symptoms, n (%)** |  |  |  |  |
| Fever | 41 (50) | 19 (57.6) | 22 (44.9) | 0.324 |
| Headache | 48 (58.5) | 23 (69.7) | 25 (51) | 0.121 |
| Neck stiffness | 7 (8.5) | 5 (15.2) | 2 (4.1) | 0.108 |
| Altered Mental Status | 33 (40.2) | 7 (21.2) | 26 (53.1) | 0.01 |
| Visual symptoms | 9 (11) | 2 (6.1) | 7 (14.3) | 0.306 |
| Auditory symptoms | 8 (9.8) | 1 (3) | 7 (14.3) | 0.137 |
| Seizures | 9 (11) | 4 (12.1) | 5 (10.2) | 1 |
| Glasgow Coma Scale within - /+3 days of the index date | 15 (10, 15) | 15 (15, 15) | 10 (8, 15) | 0.001 |
| **Lumbar puncture during hospitalization** |  |  |  |  |
| First open pressure (cmH_2_O) | 24 (16, 29.2) | 24.5 (18.9, 28) | 23.5 (16, 30.5) | 1 |
| **Profiles of CSF within -/+ 30 days of the index date** |  |  |  |  |
| Positive *Cryptococcal* antigen | 73 (88.0) | 42 (84.0) | 31 (93.9) | 0.302 |
| Positive India Ink | 66 (79.5) | 29 (87.9) | 37 (74.0) | 0.209 |
| White blood cell (/ul) | 47 (5, 156) | 10 (2, 83) | 85.5 (9.2, 261.2) | 0.003 |
| Red blood cell (/ul) | 23 (3, 155) | 16 (2, 82) | 26 (3, 162.5) | 0.514 |
| Total protein (mg/dL) | 92.8 (53.5, 189.1) | 70 (46, 125) | 136.1 (69, 228) | 0.006 |
| CSF glucose (mg/dL) | 39 (16, 53.5) | 48 (34, 58) | 27.5 (12, 47.5) | 0.009 |
| **Positive blood culture that grew *Cryptococci*** | 29 (34.9) | 18 (54.5) | 11 (22.0) | 0.005 |
| **Serum biochemical profiles within 30 days prior to the index date** |  |  |  |  |
| Positive *Cryptococcal* antigen | 61 (73.5) | 26 (78.8) | 35 (70.0) | 0.526 |
| Serum glucose (mg/dL) | 122.5 (109.8, 166.5) | 113 (98, 138) | 133 (112.8, 185.2) | 0.005 |
| ＜140 mg/dL | 50 (60.2) | 24 (72.7) | 26 (52.0) | < 0.001 |
| 140-199 mg/dL | 17 (20.5) | 6 (18.2) | 11 (22.0) |  |
| ≧200 mg/dL | 15 (18.1) | 2 (6.1) | 13 (26.0) |  |
| ESR (mm/hr) | 31 (16, 61) | 51 (19.5, 66.5) | 21 (14, 47) | 0.27 |
| White blood cell (1x10^3^/ul) | 8 (5.8, 10.9) | 6.5 (4.3, 9.1) | 9.1 (6.5, 13.8) | 0.005 |
| Creatinine (mg/dL) | 0.9 (0.7, 1) | 0.8 (0.6, 1) | 0.9 (0.7, 1.1) | 0.098 |
| Alanine aminotransferase (ALT) (IU/L) | 26.5 (18.2, 50.8) | 25 (18.8, 55) | 27 (17.5, 49) | 0.966 |
| Hemoglobin (g/dL) | 11.9 (10.1, 13.3) | 12.5 (11.7, 13.9) | 10.9 (9.7, 12.8) | 0.008 |
| CD4 / mm^3^ | 34.5 (19.5, 67.5) | 30 (17.5, 62) | 82 (55.5, 533.5) | 0.156 |
| **Medical treatment during hospitalization** |  |  |  |  |
| Amphotericin B or Liposomal Amphotericin B | 68 (81.9) | 29 (87.9) | 39 (78) | 0.394 |
| Amphotericin B | 64 (77.1) | 29 (87.9) | 35 (70) | 0.103 |
| Liposomal Amphotericin B | 20 (24.1) | 6 (18.2) | 14 (28) | 0.446 |
| Flucytosine | 60 (72.3) | 24 (72.7) | 36 (72) | 1 |
| Fluconazole | 76 (91.6) | 30 (90.9) | 46 (92) | 1 |
| Itraconazole | 1 (1.2) | 1 (3) | 0 (0) | 0.398 |
| **Surgical shunt** | 25 (30.1) | 10 (30.3) | 15 (30) | 1 |
| **Outcome** |  |  |  |  |
| **Mortality, n (%)** |  |  |  |  |
| 1-month | 14 (16.9) | 2 (6.1) | 12 (24.0) | 0.066 |
| 3-month | 22 (26.5) | 4 (12.1) | 18 (36.0) | 0.031 |
| 1-year | 38 (45.8) | 8 (24.2) | 30 (60.0) | 0.003 |
| 3-year | 45 (54.2) | 10 (30.3) | 35 (70.0) | < 0.001 |

**Abbreviations: CSF, cerebrospinal fluid; ESR, erythrocyte sedimentation rate; IQR, interquartile range.**

a Categorical variables are presented as frequency (%) and continuous variables are presented as median (IQR), if not otherwise specified.

b P-values are calculated by Kruskal-Wallis test for continuous variables and Chi-square test (or Fisher’s exact test as appropriate) for categorical variables.

c Index date was the date of positive cerebrospinal fluid culture that grew Cryptococcal species.

d Comorbidities were defined by the ICD diagnosis codes corresponding to each disease (Supplementary Table 1) that were recorded within one year prior to the index date.
